# Urinary exosomal microRNA profiling in intermediate-risk prostate cancer

**DOI:** 10.1038/s41598-021-86785-z

**Published:** 2021-04-01

**Authors:** Mee Young Kim, Hyunwoo Shin, Hyong Woo Moon, Yong Hyun Park, Jaesung Park, Ji Youl Lee

**Affiliations:** 1 Catholic Cancer Research Institute, College of Medicine, The Catholic University of Korea, Seoul, Republic of Korea; 2 Department of Urology, Seoul St. Mary’s Hospital, College of Medicine, The Catholic University of Korea, Seoul, Republic of Korea; 3 Department of Mechanical Engineering, Pohang University of Science and Technology, Pohang, Republic of Korea

**Keywords:** Cancer, Urology

## Abstract

MicroRNAs (miRNAs) of urine exosomes have emerged as biomarkers for urological cancers, owing to their high stability. MiRNAs have been linked to factors associated with aggressive prostate cancer such as biochemical recurrence (BCR) and metastasis. In this study, we aimed to identify urinary exosomal miRNAs as prognostic markers associated with BCR in intermediate-risk prostate cancer. We profiled the expression levels of miRNAs via next generation sequencing in urinary exosomes from 21 non-BCR patients and 6 BCR patients of intermediate-risk prostate cancer. A total of 21 urinary exosomal miRNAs were found to be differentially expressed (> twofold) in BCR patients compared to non-BCR patients. For external validation, we validated these results using quantitative reverse transcription PCR in an independent cohort of 28 non-BCR patients and 26 BCR patients. A validation analysis revealed that three miRNAs (miR-26a-5p, miR-532-5p, and miR-99b-3p) were upregulated in exosomes from BCR patients. The univariate and multivariate Cox regression analyses showed that miR-532-5p was an important predictive factor for BCR of intermediate-risk prostate cancer. In conclusion, miR-532-5p in urine exosomes might be a potential biomarker for predicting BCR, which is a poor prognosis in patients with intermediate-risk prostate cancer. Further research is needed on the biological functions and mechanisms of this miRNA.

## Introduction

Prostate cancer is the second most commonly diagnosed cancer in men in the world, and it is the fifth leading cause of cancer deaths^1^. Most cases of prostate cancer are clinically localized, and they are divided into low, intermediate, or high-risk groups by D'Amico's classification. Of the three groups, intermediate-risk prostate cancer is the most heterogeneous disease and reveals various oncological outcomes. More precise stratification of intermediate-risk prostate cancer is important in making optimal decisions for patients among a variety of treatment options, including radical prostatectomy (RP), radiotherapy (RT), focal therapy, and active surveillance. Therefore, there is a significant clinical need to find biomarkers for new classification systems to improve decision making in intermediate-risk prostate cancer.

Exosomes are small extracellular vesicles secreted by cells and are present in various body fluids, including blood, urine, saliva, tears, semen, breast milk, and ascites. Exosomes contain a variety of molecules that exhibit biological activity, such as proteins, lipids, and nucleic acids, and reflect the states and types of their cells of origin^2–4^. Due to these advantages, exosomes are emerging as non-invasive biomarkers for diagnosis, prognosis, and response to treatment^5^. For example, serum exosomes enriched in glycpican-1 could be used as biomarkers for pancreatic cancer^6^, and serum exosomes containing glioblastoma-specific epidermal growth factor receptor (EGFR) vIII may serve as potential biomarkers for glioblastoma^7^. Recently, the ExoDx Prostate was developed; this simple, non-invasive urine test assesses the expression of three exosomal RNAs associated with high-grade prostate cancer^8^.

MicroRNAs (miRNA) are conserved small non-coding RNAs (~ 22 nucleotides) that broadly regulate gene expression at the post-transcriptional level by targeting the 3′UTR of mRNAs^9^. In general, miRNAs are important for normal development and are involved in various biological processes^10^. The aberrant expression of miRNAs is associated with many human diseases, including cancer^11^. In addition, miRNAs are present not only within cells, but also in extracellular spaces such as blood and urine. These circulating extracellular miRNAs act as mediators of cell–cell communication and have been reported as potential diagnostic, prognostic, and predictive biomarkers in cancer^12,13^. In particular, circulating extracellular miRNAs packaged in exosomes can be more stably detected, because they are highly protected from degradation by RNase^14^. MiR-21 which is one of the representative oncogenic miRNAs was upregulated in urinary exosomes of prostate cancer patients compared to healthy controls, and it was suggested that urinary exosomal miR-21 may be a diagnostic biomarker for prostate cancer^15,16^. In another study with regard to prostate cancer, Huang et al. identified that plasma exosomal miR-1290 and miR-375 could be prognostic biomarkers for castration-resistant prostate cancer^17^.

We have recently reported that it is possible to successfully distinguish prostate cancer from benign prostatic hyperplasia (BPH) with high specificity and selectivity using the exosomes isolated from urine with the aqueous two-phase system (ATPS) method. The ATPS is an exosome isolation method that is simple and has a higher recovery efficiency than the other exosome isolation technique, ultracentrifugation, because it can separate exosomes with ~ 100% efficiency within ~ 30 min^18^. In this study, we performed miRNA profiling using urinary exosomes of prostate cancer patients with intermediate risk who underwent RP and investigated its potential as a predictive biomarker for biochemical recurrence (BCR).

## Results

### Clinical characteristics

Study subjects were selected as a discovery cohort of 27 patients and a validation cohort of 54 patients in clinically intermediate-risk prostate cancer who underwent RP. Their baseline demographics and tumor characteristics of non-BCR and BCR are described in Table 1. As shown in Table 1, all patients showed a preoperative PSA less than 20 ng/ml, a clinical Gleason score of 7, and a clinical stage of T2. There was no statistically significant difference between the non-BCR and BCR groups with respect to age, preoperative PSA, Gleason score, pathologic stage, margin status, or prostatic intraepithelial neoplasia (PIN) (p > 0.05).

**Table 1:** Clinical characteristics of patients in the discovery and validation cohort.

Variables	Discovery cohort (small RNA seq)			Validation cohort (RT-qPCR)		
	Non-BCR (n = 21)	BCR (n = 6)	*p*-value	Non-BCR (n = 28)	BCR (n = 26)	*p*-value
Mean age, years (SD)	67.8 (5.8)	69.3 (6.7)	0.616	66.6 (6.4)	65.2 (8.5)	0.500
Pre-operative PSA (ng/mL, SD)	7.9 (3.6)	8.9 (4.7)	0.670	7.7 (4.1)	6.3 (2.2)	0.101
**Gleason score, N (%)**			0.927			0.061
3 + 4	11 (52.4)	3 (50)		19 (67.9)	11 (42.3)	
4 + 3	10 (47.6)	3 (50)		9 (32.1)	15 (57.7)	
**Pathologic stage, N (%)**						
pT2a-c	21 (100)	6 (100)		28 (100)	26 (100)	
**Margin status, N (%)**			0.056			0.061
Negative	18 (85.7)	2 (33.3)		21 (75)	13 (50.0)	
Positive	3 (14.3)	4 (66.7)		7 (25)	13 (50.0)	
**PIN, N (%)**			0.755			0.769
Negative	0 (0)	0 (0)		1 (3.6)	0 (0.0)	
I	11 (52.4)	4 (66.7)		9 (32.1)	8 (30.8)	
II	9 (42.9)	2 (33.3)		13 (46.4)	14 (53.8)	
III	1 (4.8)	0 (0)		5 (17.9)	4 (15.4)	
Mean time to BCR, months (range)		31.1 (9.1–52.1)			28.5 (5.1–59.4)	

### Next generation sequencing (NGS) of urinary exosomes from prostate cancer patients

To compare the miRNA profiles of non-BCR and BCR patients, we carried out small RNA sequencing using urinary exosomes from 21 patients with non-BCR and 6 patients with BCR. Hierarchical clustering analysis showed that the samples did not cluster into specific groups (data not shown). However, a total of 21 urinary exosomal miRNAs were significantly upregulated in BCR patients compared to non-BCR ones (p-value < 0.05, fold-change > 2). Among the microRNAs with significantly increased differential expression levels, we selected 10 miRNAs as candidates due to their read counts (Table 2, Discovery cohort).

**Table 2:** Relative expression of candidate miRNAs.

Gene symbol	Discovery cohort (21 non-BCR vs 6 BCR)		Test cohort (10 non-BCR vs 9 BCR)		Validation cohort (28 non-BCR vs 26 BCR)	
	Fold change	*p*-value	Fold change	*p*-value	Fold change	*p*-value
miR-151a-3p	2.96	0.004	1.22	0.600	–	–
miR-23b-3p	2.80	0.003	1.11	0.753	–	–
miR-363-3p	2.77	0.007	1.70	0.056	1.52	0.186
miR-148a-3p	2.68	0.013	1.08	0.909	–	–
miR-200a-5p	2.55	0.001	1.20	0.702	–	–
let-7i-5p	2.32	0.001	1.21	0.691	–	–
miR-26a-5p	2.21	0.010	1.57	0.147	2.00	0.043
miR-378a-3p	2.09	0.043	1.09	0.891	–	–
miR-532-5p	2.08	0.007	1.68	0.020	2.01	0.022
miR-99b-3p	2.08	0.012	2.00	0.073	1.84	0.022

### Validation of the RNA-sequencing data by RT-qPCR analysis

**Figure 1:**
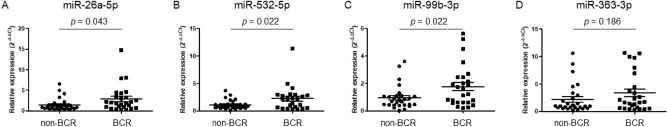
Validation of 4 miRNAs using RT-qPCR analysis. Relative expression levels of miR-26a-5p (**A**), miR-532-5p (**B**), miR-99b-3p (**C**), and miR-363-3p (**D**) in 28 non-BCR and 26 BCR prostate cancer patients. RNU6B was used as an internal control to normalize the data.

The expression levels of the 10 candidate miRNAs were analyzed in the smaller test cohort of 10 patients with non-BCR and 9 patients with BCR using RT-qPCR. The RT-qPCR results showed that miR-26a-5p, miR-532-5p, miR-99b-3p, and miR-363-3p were expressed at levels more than 1.5-fold higher in BCR patients (Table 2, Test cohort). To clarify this, the expression levels of four miRNAs in 28 patients with non-BCR and 26 patients with BCR, including previous the smaller test cohort patients, were further analyzed. Based on validation results, three miRNAs (miR-26a-5p, miR-532-5p, and miR-99b-3p) were significantly upregulated in urinary exosomes from BCR patients (Fig. 1; Table 2, Validation cohort).

### Predictive significance of exosomal miR-532-5p for prostate cancer recurrence

**Figure 2:**
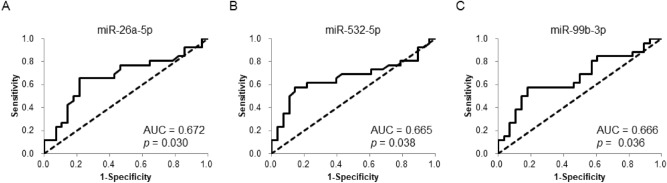
Predictive ability of urinary exosomal miRNAs. ROC curves for miR-26a-5p (**A**), miR-532-5p (**B**), and miR-99b-3p (**C**) to distinguish two sets of patients comprised of 28 non-BCR patients and 26 BCR patients.

Next, ROC curves were plotted to assess the diagnostic ability of these three miRNAs to identify BCR. The AUC values for miR-26a-5p, miR-532-5p, and miR-99b-3p were 0.672, 0.665, and 0.666, respectively, indicating the potential to distinguish BCR from non-BCR cases (Fig. 2).

**Figure 3:**
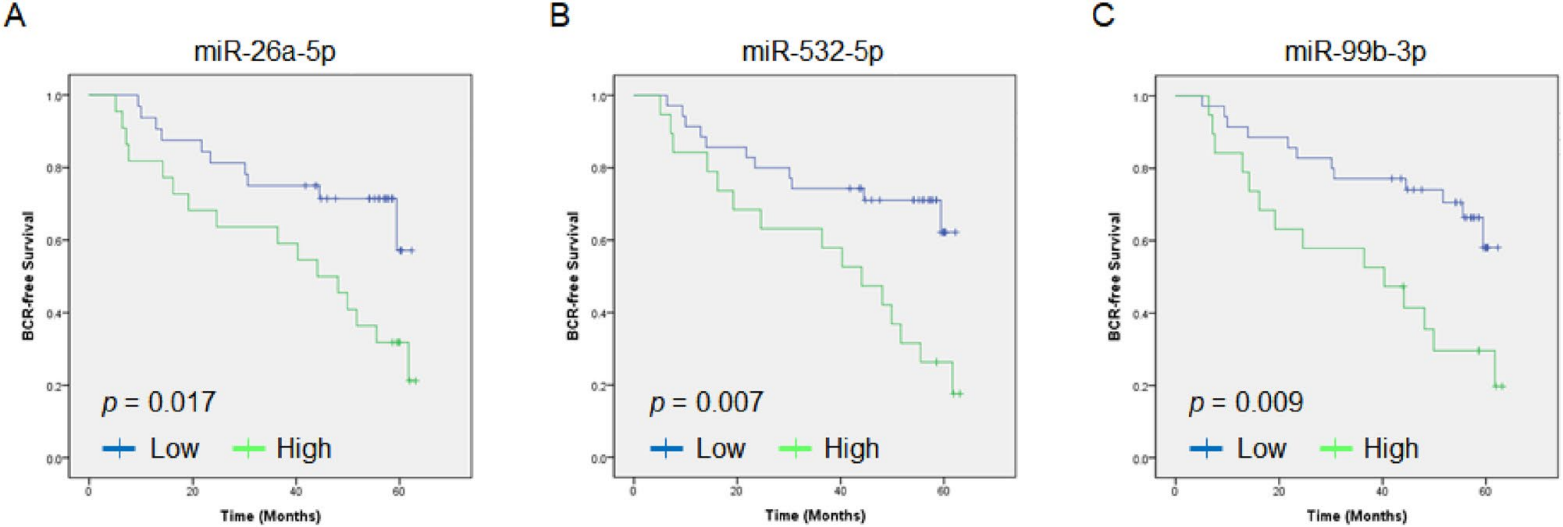
Comparison of BCR-free survival of the prostate cancer patients with exosomal miR-26a-5p (**A**), miR-532-5p (**B**), and miR-99b-3p (**C**). Patients with high miRNA expression showed significantly shorter BCR-free survival than those with low miRNA expression.

To assess the clinical significance of miRNA expression, a BCR-free survival assay was performed on the validation cohort of 54 patients. Kaplan–Meier analysis showed that patients with high three miRNA (miR-26a-5p, miR-532-5p, and miR-99b-3p) expression in their urinary exosomes had significantly shorter BCR-free survival after RP than patients with low miRNA expression did (Fig. 3).

Cox regression analyses were performed using the following covariates: age, pre-operative PSA levels, Gleason grade, margin status, prostatic intraepithelial neoplasia (PIN), and relative exosomal miRNA expression levels. In univariate analysis, miR-532-5p and miR-99b-3p were found to be the significant factors for BCR (HR: 2.01 [95% CI 1.13–3.58] and 1.87 [1.06–3.29], respectively), while age, pre-operative PSA, Gleason grade, margin status, PIN, and miR-26a-5p expression were not. In multivariate analysis, miR-532-5p expression was the only significant factor for prostate cancer recurrence (HR: 2.10 [95% CI 1.15–3.81]) (Table 3).

**Table 3:** Univariate and multivariate Cox-regression analyses of prostate cancer recurrence.

Variables	Univariate		Multivariate	
	HR (95% CI)	*p*-value	HR (95% CI)	*p*-value
Age	0.98 (0.91–1.06)	0.6327	–	–
Pre-operative PSA	0.89 (0.75–1.06)	0.2007	–	–
Gleason score	1.46 (0.65–3.28)	0.3603	–	–
PIN	1.12 (0.54–2.36)	0.7587	–	–
Margin status	3.00 (0.95–9.48)	0.0612	3.26 (0.93–11.40)	0.0645
Relative miR-26a-5p expression	1.38 (0.99–1.93)	0.0586	–	–
Relative miR-532-5p expression	2.01 (1.13–3.58)	0.0182	2.10 (1.15–3.81)	0.0153
Relative miR-99b-3p expression	1.87 (1.06–3.29)	0.0314	–	–

## Discussion

Intermediate-risk prostate cancer remains a difficult disease for absolute prognostication, as it consists of a heterogeneous population of patients with variable prognoses. The biochemical and clinical recurrence rates of these patients after RP, RT, or brachytherapy vary from 2 to 70%, and so there has been increasing effort to develop prognostic biomarkers to reflect the actual risk of recurrence more accurately. In fact, various studies have been conducted to identify biomarkers that are able to predict BCR after RP precisely at the DNA, RNA, and protein levels^19,20^. Wei et al. showed that miR-1 in prostate cancer tissue, a novel miRNA-based BCR biomarker, is a potential predictive factor^21^. In addition, Zhao et al. suggested 5 miRNAs (miR-30c-5p, miR-31-5p, miR-141-3p, miR-148a-3p, and miR-221-3p) as a tissue-based multiple miRNA panel for prognostic biomarkers^22^. The BCR biomarkers reported to date are mainly drawn from studies using tissues obtained after RP, but it is difficult to use easily as a testing tool because there are several disadvantages to obtaining the tissue. In contrast, urine is a good tool for biomarker testing because it can be easily obtained in a non-invasive manner.

Our study aimed to investigate urinary exosomal miRNA as a potential predictive biomarker for BCR after RP in intermediate-risk prostate cancer patients. We found 21 upregulated miRNAs in the urinary exosomes of BCR patients compared to the urinary exosomes of non-BCR patients. Next, we validated 3 upregulated miRNAs in independent cohorts using RT-qPCR, and finally miR-532-5p, a single miRNA, was identified as a marker for predicting BCR after RP. To the best of our knowledge, this is the first study to identify miRNA as a predictive biomarker for recurrence using NGS in the urinary exosomes of patients at intermediate risk. Bryant et al. reported that miR-375 and miR-141 are increased in both serum exosomes and microvesicles of recurrent prostate cancer patients compared with non-recurrent prostate cancer patients following RP^23^. Fredsøe et al. found urine-based miRNA biomarkers that could accurately predict the likelihood of recurrence after RP. This three-miRNA model (miR-125b-5p*let-7a-5p/miR-151-5p) significantly predicted time to BCR independently of routine clinicopathological variables such as PSA, clinical T stage, and Gleason score^24^. Although this was also a study based on urinary exosomes, our study has some differences. The exosome was effectively isolated using the ATPS method, providing high recovery efficiency compared to the conventional method, and we successfully performed miRNA profiling in the urinary exosome using NGS. Moreover, we identified a single miRNA that could discriminate between non-BCR and BCR patients.

MiR-532-5p has been reported to be involved in tumorigenesis in various cancers, and interestingly, it has both oncogenic and tumor suppressor roles, depending on the type of cancer. For instance, miR-532-5p was upregulated in breast cancer tissues compared with normal tissues, and the downregulation of miR‑532‑5p decreased the cell proliferation and migration of breast cancer cells^25^. For colorectal cancer (CRC), miR-532-5p was increased in CRC tissues, and furthermore, miR‑532‑5p overexpression promoted CRC cell growth^26^. The results of these studies indicated that miR-532-5p might be an oncogenic miRNA. Meanwhile, miR-532-5p was downregulated in renal cell carcinoma (RCC) tissue and cell lines. Moreover, miR-532-5p not only inhibited RCC cell proliferation in vitro, but it also suppressed tumorigenicity in vivo^27^. In addition, the expression of miR-532-5p was decreased in bladder cancer, and loss of miR-532-5p promoted the proliferation and invasion of bladder cancer cells^28^. These findings suggested that miR-532-5p functions as a tumor suppressor. However, the role of miR-532-5p in prostate cancer remains unknown. Therefore, further study is required to clarify the roles of miR-532-5p in prostate cancer.

Our study has some limitations. First, this study had a relatively small sample size, with samples collected from a single center, and it lacks external validation. Second, we have proposed that miR-532-5p is a predictor of prostate cancer recurrence in urinary exosomes, but the mechanism associated with this miRNA is still unclear in prostate cancer. Therefore, further study is needed on the roles and related mechanisms of miR-532-5p.

## Materials and methods

### Patients

The study protocol was approved and carried out in accordance with the approved guidelines by the Institutional Review Board at the Catholic University of Korea, Seoul St. Mary’s Hospital (IRB approval No. KC14SISI0213). Urine samples of clinically localized prostate cancer adult’s patients who underwent RP were obtained from the Korea Prostate Bank (Seoul, Republic of Korea) with informed consent. This included 49 patients with non-BCR and 32 patients with BCR. BCR was defined as an increase in PSA level to ≥ 0.2 ng/mL after RP. This study was approved by the Institutional Review Board of the Catholic University of Korea, College of Medicine. The characteristics of the recurrent and non-recurrent patients are summarized in Table 1.

Urine was catheterized during RP, and was centrifuged at 2500 rpm for 20 min at 4 °C. The supernatant was transferred to new tubes and stored at − 80 °C.

### Urinary exosome isolation

Urinary exosomes were isolated using ATPS (Exo2D, EsosomePlus, Seoul, Republic of Korea) according to the manufacturer’s instructions. Briefly, samples were thawed at 4 °C and then were centrifuged again at 2500 rpm for 5 min to remove any residual cell debris. Next, 10 mL of the supernatant were transferred to 15-mL tubes for exosome preparation. Then, 2 mL of Exo2D reagent B were added to the clarified urine and the mixture was incubated at 4 °C for 30 min. The precipitated exosomes were recovered by centrifugation at 3000×*g* for 30 min at 4 °C. Exosome pellets were resuspended with 200 μL of phosphate-buffered saline.

### Exosomal RNA extraction

Exosomal total RNA was extracted from the urinary exosomes using a miRNeasy Serum/Plasma Kit (Qiagen, Hilden, Germany)^29^. The urinary exosomes were disrupted and homogenized in 1 mL of QIAzol lysis reagent, and the rest of the procedure was performed according to the manufacturer’s protocol. The extracted RNA was eluted with 14 µL of RNase-free water. The quantity and purity of the RNA was then measured using a NanoDrop 2000 spectrophotometer system (Thermo Fisher Scientific, Waltham, MA, USA). The RNA integrity was assessed by an Agilent 2100 bioanalyzer using the RNA 6000 Pico Chip (Agilent Technologies, Amstelveen, The Netherlands).

### Library preparation and sequencing

For control and test RNAs, the construction of a library was performed using a NEBNext Multiplex Small RNA Library Prep kit (New England BioLabs, Inc., Ipswich, MA, USA) according to the manufacturer’s instructions^30^. Briefly, for library construction, total RNA from each sample was used at 1 µg to ligate the adaptors and then cDNA was synthesized using reverse-transcriptase with adaptor-specific primers. PCR was performed for library amplification, and library clean-up was carried out using a QIAquick PCR Purification Kit (Qiagen) and AMPure XP beads (Beckman Coulter, Inc., Brea, CA, USA). The yield and size distribution of the small RNA libraries were assessed by the Agilent 2100 Bioanalyzer instrument for the High-Sensitivity DNA Assay (Agilent Technologies). High-throughput sequences were produced by a NextSeq 500 system as way of single-end 75 sequencing (Illumina, San Diego, CA, USA).

### Data analysis

Sequence reads were mapped by the Bowtie 2 software tool in order to obtain BAM files (alignment files). A mature miRNA sequence was used as a reference for mapping. Read counts mapped on the mature miRNA sequence were extracted from the alignment file using bedtools (v2.25.0) and Bioconductor^31^, which uses the R (version 3.2.2) statistical programming language (https://www.r-project.org/). Read counts were used to determine the expression levels of the miRNAs. The quantile normalization method was used for comparisons between samples.

### Reverse transcription-quantitative polymerase chain reaction (RT-qPCR)

All of the miRNA specific probes used (TaqMan MicroRNA Assays) are commercially available from Thermo Fisher Scientific: catalog number # 4427975 (hsa-miR-26a-5p, Assay ID: 000405; hsa-let-7i-5p, Assay ID: 002221; hsa-miR-200a-5p, Assay ID: 001011; hsa-miR-532-5p, Assay ID: 001518; hsa-miR-148a-3p, Assay ID: 000470; hsa-miR-23b-3p, Assay ID: 000400; hsa-miR-99b-3p, Assay ID: 002196; hsa-miR-363-3p, Assay ID: 001271; hsa-miR-378a-3p, Assay ID: 001314; hsa-miR-151a-3p, Assay ID: 002254; hsa-miR-191-5p, Assay ID: 002299; RNU6B, Assay ID: 001093).

The RT reactions were performed using the TaqMan MicroRNA Reverse Transcription Kit (Thermo Fisher Scientific) following the manufacturer’s protocol. The qPCR analysis was performed on a StepOne Plus real-time thermocycler using Taqman Universal Master Mix II with no UNG (Thermo Fisher Scientific). Relative miRNA expression levels were determined by normalizing to RNU6B using the 2^−ΔΔCT^ method.

### Statistical analysis

Statistical analysis was carried out with GraphPad Prism 5 (GraphPad Software, CA, USA) and SPSS 22.0 (IBM, NY, USA). We utilized receiver operating characteristic (ROC) curve analysis to estimate the diagnostic values of the urinary exosomal miRNAs.
